# Gradient-based delineation of the primary GTV on FLT PET in squamous cell cancer of the thoracic esophagus and impact on radiotherapy planning

**DOI:** 10.1186/s13014-014-0304-5

**Published:** 2015-01-09

**Authors:** Guifang Zhang, Dali Han, Changsheng Ma, Jie Lu, Tao Sun, Tonghai Liu, Jian Zhu, Jingwei Zhou, Yong Yin

**Affiliations:** Department of Radiation Physics, Shandong Cancer Hospital and Institute, Shandong’s Key Laboratory of Radiation Oncology, Jiyan Road 440, Jinan, 250117 Shandong Province P. R. China; Department of Radiation Oncology, Shandong Cancer Hospital and Institute, Shandong’s Key Laboratory of Radiation Oncology, Jinan, China; Department of Radiology, Shandong Cancer Hospital and Institute, Jinan, 250117 Shandong Province P. R. China

**Keywords:** Esophageal carcinoma, Radiotherapy, FLT-PET, GTV delineation

## Abstract

**Background:**

To validate a gradient-based segmentation method for gross tumor volume(GTV) delineation on ^8^F-fluorothymidine (FLT)positron emission tomography (PET)/ computer tomography (CT) in esophageal squamous cell cancer through pathologic specimen, in comparison with standardized uptake values (SUV) threshold-based methods and CT. The corresponding impact of this GTV delineation method on treatment planning was evaluated.

**Methods and materials:**

Ten patients with esophageal squamous cell cancer were enrolled. Before radical surgery, all patients underwent FLT-PET/CT. GTVs were delineated by using four methods. GTV_GRAD_, GTV_1.4_ and GTV_30%max_ were segmented on FLT PET using a gradient-based method, a fixed threshold of 1.4 SUV and 30% of SUV_max_, respectively. GTV_CT_ was based on CT data alone. The maximum longitudinal tumor length of each segmented GTV was compared with the measured tumor length of the pathologic gross tumor length (L_Path_). GTV_GRAD_, GTV_1.4_ and GTV_30%max_ were compared with GTV_CT_ by overlap index. Two radiotherapy plannings (plan_GRAD_) and (plan_CT_) were designed for each patient based on GTV_GRAD_ and GTV_CT_. The dose-volume parameters for target volume and normal tissues, CI and HI of plan_GRAD_ and plan_CT_ were compared.

**Results:**

The mean ± standard deviation of L_Path_ was 6.47 ± 2.70 cm. The mean ± standard deviation of L_GRAD_,L_1.4_, L_30%max_ and L_CT_ were 6.22 ± 2.61, 6.23 ± 2.80, 5.95 ± 2.50,7.17 ± 2.28 cm, respectively. The Pearson correlation coefficients between L_Path_ and each segmentation method were 0.989, 0.920, 0.920 and 0.862, respectively. The overlap indices of GTV_GRAD_, GTV_1.4_, GTV_30%max_ when compared with GTV_CT_ were 0.75 ± 0.12, 0.71 ± 0.12, 0.57 ± 0.10, respectively. The V_5_, V_10_, V_20_, V_30_ and mean dose of total-lung,V_30_ and mean dose of heart of plan_GRAD_ were significantly lower than plan_CT_.

**Conclusions:**

The gradient-based method provided the closest estimation of target length. The radiotherapy plannings based on the gradient-based segmentation method reduced the irradiated volume of lung, heart in comparison to CT.

## Introduction

Radiotherapy (RT) is one of the most important treatment modalities for esophageal cancer. CT has been the standard of GTV delineationn in esophageal cancer, however, there is growing interest in using PET-guided GTV delineation [1] to take advantage of increased contrast between tumor and surrounding normal tissue.

^18^F-fluorodeoxyglucose (FDG) is the most frequently used radiopharmaceutical for oncologic PET scanning. However, FDG-PET has limitations in specificity. False-positive results can be caused by inflammation, including peritumoural inflammatory reactions [2]. Studies have shown that FLT is a good imaging tracer for cell proliferation [3-6] and tumor volume change [7]. However, little data is available on the clinical use of FLT-PET in various carcinomas [8-10], including esophageal cancer.

A number of methods have been used for GTV delineation in PET including manual contouring [11,12] and semi-automatic threshold-based segmentation [13,14]. However, these methods have inherent limitations. To overcome these limitations gradient-based segmentation method has been developed to identify the tumor based on changes in count levels at the tumor border. This method has been validated using phantoms [15,16] and through comparison to pathologic specimens for head and neck carcinoma [15] and non-small-cell lung cancer (NSCLC) [17]. However, data for GTV delineation in esophageal cancer using gradient-based segmentation is lacking and in particular using FLT-PET. Our goal in this current study was to validate a gradient-based segmentation method for GTV delineation in esophageal cancer through pathologic specimens in comparison with SUV thresholds and CT. The impact of using the gradient-based delineated GTV in comparison to the CT delineated GTV was evaluated using dose volume parameters of the lung, heart, and spinal cord.

## Methods and materials

### Patients selection

Ten patients (mean age 60 years, range 52-75) with histologically proven esophageal squamous cell carcinoma were prospectively enrolled in this study between September 2008 and January 2009. Four patients had middle thoracic esophageal cancer, and six had lower thoracic disease. None of the patients had previously been treated with preoperative chemotherapy or RT. First, all patients underwent routine pretreatment evaluation, including physical examination, complete blood count, biochemistry surveys of liver and kidney function, chest radiographs, electrocardiograms, barium esophagograms, esophagogastroscopy with tumor biopsy, ultrasound evaluation of the neck and abdomen, and pulmonary function testing. Informed consent was obtained from all patients.

### Image acquisition

FLT-PET/CT image acquisitions were performed within 4 days prior to surgical tumor resection with a dedicated PET/CT scanner (Discovery LS, GE Healthcare). The patients fasted for at least 6 hours and rested for 15 minutes before the injection of 300–400 MBq of FLT. Images were obtained 60 min after injection. The scans were performed for 5 min/ bed position from head to femur, each covering 14.5 cm, at an axial sampling thickness of 4.25 mm/slice. Both PET and CT acquisition were performed in free respiration. Data were reconstructed using an iterative reconstruction technique and attenuation correction derived from CT data. The CT,PET and fused PET/CT images were transmitted to MIM Maestro (MIM Software Inc, Cleveland, OH) for GTV delineation.

### Delineation of GTV

**Figure 1:**
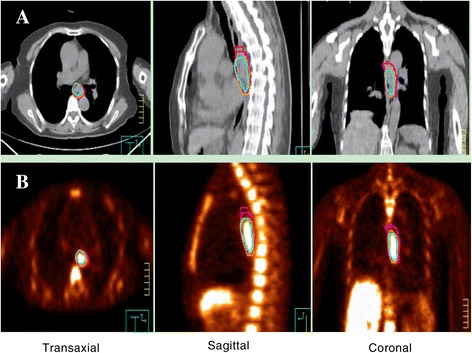
**Overview of the tumor delineation for one patient with middle thoracic esophageal cancer. (A)** Gross tumor lengths delineated on CT images by different methods. **(B)** Gross tumor lengths delineated on FLT PET/CT images by different methods. Volumes are displayed in transaxial, sagittal and coronal planes. Purple, orange, blue and Red contours illustrate different parameters (e.g. GRAD, 1.4 SUV threshold, 30% of SUV_max_ and CT).

For each patient, four different GTVs were generated (Figure 1). The PET/CT images were reviewed by one experienced nuclear medicine physician and a radiation oncologist, GTV_GRAD_, GTV_1.4_ and GTV_30%max_ were automatically segmented on PET images using the gradient-based method, fixed threshold values at 1.4 SUV and 30% of SUV_max_. The longitudinal gross tumor length of GTV_GRAD_, GTV_1.4_ and GTV_30%_max, were recorded as L_GRAD,_ L_1.4_ and L_30%max_, respectively. One experienced radiologist and one radiation oncologist, both unaware of the results from PET or surgery, segmented the CT images (GTV_CT_), using esophageal wall thickness of 5 mm or more and esophageal wall diameter without gas more than 10mm as the criteria of primary tumor. The longitudinal gross tumor length of CT was measured and recorded as L_CT_. Figure 1 shows case that overview of the tumor delineated by different methods. The 1.4 SUV threshold was chosen based on a previous study [7] and the 30% of SUV_max_ was based on clinical experience. The method for gradient-based segmentation has been previously described. “It calculates spatial derivatives along the tumor radii and then defines the tumor edge on the basis of derivative levels and continuity of the tumor edge. The software relies on an operator- defined starting point near the center of the lesion. As the operator drags out from the center of the lesion, 6 axes extend out, providing visual feedback for the starting point of gradient segmentation. Spatial gradients are calculated along each axis interactively, and the length of an axis is restricted when a large spatial gradient is detected along that axis. The 6 axes define an ellipsoid that is then used as an initial bounding region for gradient detection [16]”. The fixed SUV threshold methods involved placing a user-defined sphere over the tumor and applying the threshold inside of the sphere to segment the tumor.

### Processing of the surgical specimen

All patients underwent transthoracic esophagectomy with conventional two-or three-field lymphadenectomy. The specimens were flattened within 30 min after surgical resection, stretched to same length as measured in-situ, pinned on a plastic foam-board, then soaked and fixed with 10% formaldehyde. The specimens were cut into 5mm-wide strips of tissue following longest dimension of the tumor (including upper and lower margin)to create the pathological sections [7]. L_Path_ is defined as the pathological gross tumor length as measured under a low magnification microscope.

### GTV data analysis

The longitudinal gross tumor length and volumes of GTVs derived from different delineation methods were compared.

The mean and standard deviation of longitudinal gross tumor length for each of the segmentation method was calculated. L_GRAD_, L_1.4_ and L_30%max_ were compared with L_Path_ by means of the correction coefficients. The L_path_ were defined as the “gold standard,” but because the correction coefficient does not allow preference between GTV_PET_ and GTV_CT_, an overlap index (OI) was also calculated for each set of contours according to the expression:

$$ \mathrm{O}\mathrm{I}=\frac{{\mathrm{GTV}}_{\mathrm{PET}}\cap {\mathrm{GTV}}_{\mathrm{CT}}}{{\mathrm{GTV}}_{\mathrm{CT}}} $$

The OI reflects the inclusion of GTV_PET_ within GTV_CT_ [18].

### Treatment planning design and evaluation

**Figure 2:**
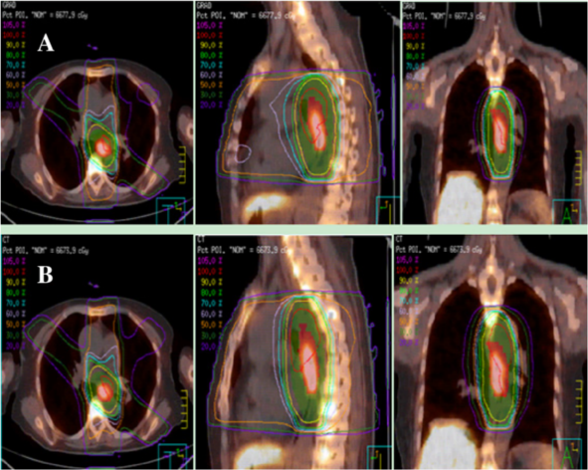
**Treatment planning simulated with FLTPET/CT for 67-years-old female patient with middle thoracic esophageal cancer. (A)** FLT PET/CT-based five-beam conformal radiation therapy **(B)** CT-based five-beam conformal radiation therapy.

Both PET and CT data for all patients were transmitted to a Pinnacle^3^ treatment planning station (Philips, 8.0 m). The clinical target volume (CTV) was created using a 3 cm margin in the craniocaudal direction (following the course of the esophagus) and a 1 cm margin in the lateral and anteroposterior directions beyond GTV_GRAD_ and GTV_CT_. The CTV was then expanded in all directions by 0.5 cm to create the planning target volume (PTV): PTV_GRAD_ and PTV_CT_. Two treatment plannings, plan_GRAD_ and plan_CT_ were designed based on PTV_GRAD_ and PTV_CT_, respectively. The radiation dose was prescribed as 60Gy in 30 fractions using a five-beam conformal radiotherapy treatment. The treatment plannings of a 67-years-old female patient with middle thoracic esophageal cancer were showed in Figure 2. The plans were designed to meet the following treatment planning goals: >95% of the PTV covered by the prescription isodose line, V_20_ of total lung < 30%, V_40_ of heart < 30% and D_max_ of spinal cord < 45 Gy.It was required that there was no cold spot in the PTV and no hot spot in esophageal wall. Cumulative DVHs were generated for evaluation and comparison of plan_GRAD_ and plan_CT_. For PTV, the values of HI (heterogeneity index) and CI (conformal index) were compared. The mean lung dose (MLD), V_5_ (volume included by 5Gy isodose curve), V_10_, V_20_, V_30_ and mean lung dose of total lung, V_30_ (volume included by 30Gy isodose curve), V_40_, mean heart dose (MHD) of heart and maximum dose of spinal cord (D_max_) were recorded.

### Statistical analysis

The Statistical Package for Social Sciences, version 13.0 (SPSS, Chicago, IL) was used for statistical analysis. The two-tailed paired Student’s *t*-test was applied to assess the differences between groups and Pearson’s correlation were performed to assess the relationship between groups. A p value of <0.05 was considered statistically significant. HI = (D_2_-D_98_)/prescription dose * 100% [19]; and CI = Vt,ref/Vt * Vt,ref/Vref [20]. [Note: Vt = target volume, Vt,ref = target volume wrapped by reference isodose, Vref = all volume wrapped by reference isodose]. CI values range beetween 0 and 1 with a value closer to 1 represents a better conformity.

## Results

**Figure 3:**
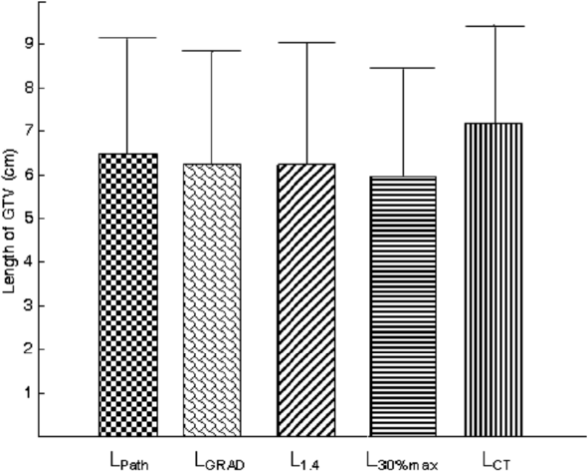
**The mean Length obtained using different methods.** Error bars indicate standard deviation.

The mean of L_Path_ was (6.47 ± 2.7) cm (range, 3.44-11.00 cm). Of the 10 primary cancers, 4 were >5 cm and 6 were <5 cm. The mean length obtained using each segmentation method was depicted in Figure 3. The correlation coefficients of L_GRAD_, L_1.4_, L_30%max_, L_CT_ with L_Path_ were 0.989 (P < 0.05), 0.920 (P < 0.05), 0.920 (P < 0.05), 0.862 (P < 0.05), respetively. In comparison to the PET determined GTV, L_CT_ led to a large over estimation of L_Path_. L_GRAD_ seemed to more greatly approximate to L_Path_.

**Figure 4:**
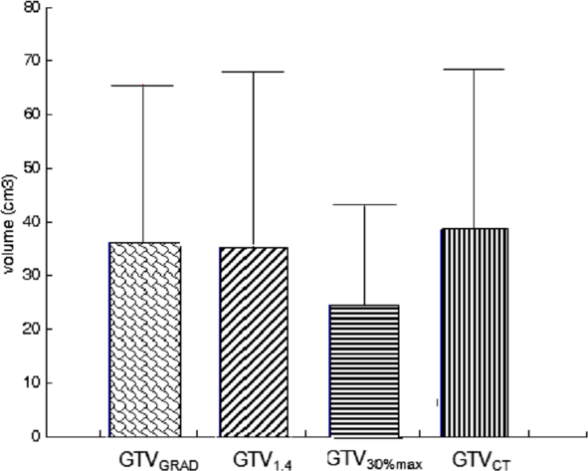
**The mean volume obtained using different methods.** Error bars indicate standard deviation.

The mean volume of GTV_CT_ was (38.37 ± 30.04) cm^3^ (range: 14.41-105.11 cm^3^),the mean volume obtained using each segmentation method was depicted in Figure 4. The GTVs generated from FLT PET/CT were significantly smaller than GTV_CT_ (P < 0.05). The mean OI of GTV_GRAD_, GTV_1.4_ and GTV_30%max_ with GTV_CT_ were 0.75 ± 0.12, 0.71 ± 0.12 and 0.50 ± 0.10, the OI of GTV_GRAD_ with GTV_CT_ was higher than GTV_1.4_, but the difference between GTV_GRAD_ and GTV_1.4_ was not significant.

Next, two RT simulation plannings using gradient-based segmentation method on FLT PET/CT and CT delineating target volumes in the Philips Pinnacle^3^ treatment planning system were compared. All the treatment plannings met the criteria that >95% of the PTV were covered by the prescription isodose line and the global hotspot was <10%. In Tables 1 and 2 the evaluation factors of PTV_GRAD_ and PTV_CT_ were listed. The difference in CI, HI, V_40_ of heart and D_max_ of spinal cord between plan_GRAD_ and plan_CT_ were not significantly different. However, the V_5_,V_10_,V_20_, V_30_ and mean dose of total-lung, V_30_ and mean dose of heart of plan_GRAD_ were significantly lower than plan_CT_.

**Table 1:** **Comparison of CI and HI of two group plannings**

**Parameter**	**plan**_**GRAD**_	**plan**_**CT**_	***t***	***P***
CI	0.75 ± 0.06	0.74 ± 0.06	0.94	0.38
HI	1.13 ± 0.02	1.14 ± 0.03	−1.16	0.28

**Table 2:** **Comparison of FLT and CT-based overall values of measured dose–volume histogram-based evaluation factors for esophageal cancer patients**

**Mean ± SD**	**plan**_**GRAD**_	**plan**_**CT**_	***t***	***P***
Total-lung				
*V*_5_ (%)	47.9 ± 15.30	56.1 ± 13.80	−6.31	0.000
*V*_10_ (%)	33.8 ± 8.90	40.1 ± 9.10	−6.78	0.000
*V*_20_ (%)	18.2 ± 10.00	23.8 ± 9.70	−4.36	0.003
*V*_30_ (%)	5.49 ± 4.40	8.9 ± 4.70	−3.17	0.016
MLD (Gy)	9.82. ± 319.50	10.96 ± 3.02	−4.70	0.002
Heart				
*V*_30_ (%)	37.6 ± 19.30	44.1 ± 20.80	−5.97	0.000
*V*_40_ (%)	20.8 ± 13.50	22.8 ± 14.10	−1.19	0.272
MHD (Gy)	22.63 ± 10.25	25.37 ± 10.71	−5.92	0.000
D_max_ of spinal cord (Gy)	44.26 ± 1.36	44.69 ± 0.29	−0.94	0.380

## Discussion

The developments of functional imaging techniques such as PET/CT have brought biologically-guided IMRT to the forefront of radiotherapy. Biologically-guided intensity-modulated radiation therapy (IMRT) has shown the advantages over traditional anatomically-based IMRT, in terms of GTV contouring, treatment planning optimization, and prognosis determination [21]. PET has been shown to supplement the information lacking in anatomical images and improves the accuracy of target volume segmentation [22]. FDG-PET image has shown the capability to accurately determine the length of esophageal tumors and GTV delineation more accurate, and improving treatment planning compared to anatomical imaging [23]. While FDG-PET has long been the most used tracer for biologic tumor imaging, in recent years, FLT has garnered attention due to its ability to characterize tumor proliferation.

In our study, we evaluated the accuracy of a gradient-based segmentation method for GTV delineation with FLT-PET using pathologically determined EC length as the gold standard. SUV threshold methods and CT visual delineation were used for comparison. Additionally we explored the feasibility of the use of FLT-PET with gradient-based segmentation for treatment planning. While all segmentation methods were found to correlate well with L_Path_, the gradient-based results were the most consistent with the pathologic results. Werner-Wasik M et al [16] demonstrated similar results for their gradient-based segmentation method when they found their method was more accurate than SUV thresholds and CT visual delineation for NSCLC when compared to a volumetric pathology gold standard.

Common methods of GTV segmentation for PET/CT images included visual judgement and SUV thresholds. Visual judgement relies solely on the visual discrimination of tumor boundaries, which is subjective and leads to inter observer bias. In addition, differences in window and level contrast settings contribute to variability [22]. Threshold methods often use a fixed SUV or a certain percentage of the maximum SUV as the threshold [23]. These methods are limited with regard to variations in tumor size which affect image count levels due to partial volume effects, variable tumor to background activity, and heterogeneous tracer uptake in the tumor. Gradient-based methods use the maximum spatial gradient to detect boundaries between lesions and normal tissue [24], which is not affected by different imaging equipments, reconstruction algorithms and sphere diameter effects [16]. It overcomes the limitations of visual judgement and SUV threshold methods.

The gradient and 1.4 SUV threshold methods had tumor lengths that were the most closely related to pathologically determined tumor lengths acquisition, and reconstruction variables that can significantly affect SUV measurements in typical less controlled clinical situations [1]. These factors would also impact the size of lesions detected with the absolute 1.4 SUV threshold, but do not affect the gradient –based measurement of lesion size which is based on detecting the maximum change in SUV levels regardless of their absolute value.

The CT segmentation result was found to overestimate the tumor length compared to pathology. This coincides with the results of Konski et al. [25], in which 25 esophageal cancer patients were scanned using CT and PET/CT. In their study the PET/CT scans showed a mean tumor length of 5.4 cm compared to 6.77 cm from CT, representing a statistically significant difference.

The CT-based contours also were found to be larger in size than the gradient-based contours, which may be due to the fact that the entirety of the esophageal wall is typically included in the CT-defined GTV, regardless of which part of the wall the tumor is in. Since the target volume shown in FLT-PET images represents FLT uptake activity, it could include the entire esophageal wall or just part of the wall. In addition, inflammation will show a thickened esophageal wall in CT images, whereas this may not affect the uptake activity shown in FLT PET images.

3DCRT plans using PTV_GRAD_ and PTV_CT_ were designed to compare dosimetric differences between treatment plannings using target volumes delineated with gradient-based and CT-based methods respectively. As shown, GTV_GRAD_ was smaller in volume than GTV_CT_, and the outer borders caused even greater differences in PTV volume, which has a direct impact on the dose given to normal tissue. While conformality and homogeneity of the two groups of PTVs were similar, plan_GRAD_ resulted in significantly lower organ at risk doses than plan_CT_. To some extent, this would reduce the side effects of radiotherapy, thereby improving patient quality of life and prognosis. However, it has also been reported in the literature that FLT PET/CT will miss some small primary tumors [26], so a variety of imaging modalities should be combined with caution in clinical practice.

The major limitation of our study is that the GTV information on the specimens was not obtained due to some technical reasons. The esophagus is an organ composed of musculature and lumen; the esophageal specimen will contract and deform after being resected, including the tumor [27]. Therefore, the volume measured on a specimen may not reflect their real values in situ. We measured the length of the specimen in situ and pinned the specimen to a flat board before fixing it with 10% formaldehyde, hoping this method would be less influenced by the deformation of esophageal lumen and thus provide an approximate estimation. Another limitation of the present study was the limited number of patients, to evaluate whether the integration of FLT PET/CT into radiotherapy of esophageal cancer can actually improve the treatment outcomes, a larger sample prospective investigation with data of treatment failures after radiotherapy planning based on PET/CT is worthy of expectation.

## Conclusions

Gradient-based segmentation provides a more precise method for esophageal cancer delineating than SUV thresholds or CT visual delineation using FLT-PET/CT, laying the foundation for future development in FLT-PET/CT and BTV-based treatment planning of esophageal cancer. Further research needs to be done to validate this work using larger sample sizes and assessing the clinical impact of treatment plannings designed using gradient-defined biologic tumor volumes.

## Abbreviations

RT: Radiotherapy
GTV: Gross target volume
CTV: Clinical target volume
PTV: Planning target volume
PET: Positron emission tomography
CT: Computer tomography
FDG: ^18^F-fluorodeoxyglucose
FLT: ^18^F-fluorothymidine
NSCLC: Non-small-cell lung cancer
IMRT: Intensity-modulated radiation therapy
SUV: Standardized uptake values
OI: Overlap index
MLD: Mean lung dose
CI: Conformal index
HI: Heterogeneity index
